# Effect of Vitamin E on Serum Levels of Vascular Endothelial Growth Factor and Angiopoietin-1 in Women with Polycystic Ovary Syndrome: A Pilot Randomized, Placebo-Controlled Trial 

**DOI:** 10.22074/IJFS.2020.45677

**Published:** 2021-01-27

**Authors:** Shabnam Shirazi, Bahram Pourghassem Gargari, Azimeh Izadi, Shiva Taghizadeh, Marziyeh Parizad

**Affiliations:** 1 Department of Biochemistry and Diet Therapy, Faculty of Nutrition and Food Sciences, Tabriz University of Medical Sciences, Tabriz, Iran; 2 Student Research Committee, Tabriz University of Medical Sciences, Tabriz, Iran; 3 Nutrition Research Center, Department of Biochemistry and Diet Therapy, Faculty of Nutrition and Food Sciences, Tabriz University of Medical Sciences, Tabriz, Iran; 4 Women’s Reproductive Health Research Center, Tabriz University of Medical Sciences, Tabriz, Iran

**Keywords:** Angiopoietins, Basic Fibroblast Growth Factor, Polycystic Ovary Syndrome, Vascular Endothelial Growth Factor, Vitamin E

## Abstract

**Background:**

Angiogenesis disturbances are common in women with polycystic ovary syndrome (PCOS). Vitamin E has antiangiogenic properties. Data on the effects of vitamin E on angiogenesis in PCOS is limited, so the current study was conducted to evaluate its effects on angiogenic indices in PCOS patients.

**Materials and Methods:**

This randomized, double-blind, placebo-controlled trial was performed on 43 women aged 20-40 years, diagnosed with PCOS (Rotterdam criteria). It was performed at the referral clinic affiliated to Tabriz University of Medical Sciences, Tabriz, Iran, from April 2017 to September 2017. Patients were randomly assigned into two groups to receive either 400 IU/day vitamin E -as alpha tocopheryl acetate- (n=22) or placebo (n=21), for 8 weeks. Anthropometric, and angiogenic parameters including body weight, fat mass and fat free mass, vascular endothelial growth factor (VEGF), basic fibroblast growth factor (bFGF), angiopoietin-1 (Ang- 1), and angiopoietin-2 (Ang-2) were measured by standard methods at the beginning and at the end of study. Statistical Package for Social Science version 25 was used for statistical analysis and P<0.05 were considered significant.

**Results:**

After adjusting for potential confounders, we observed that vitamin E supplementation significantly reduced body weight, fat mass, Ang-1, Ang-1/Ang-2 ratio and VEGF (P<0.01). We did not observe any considerable effect for vitamin E on Ang-2 level or bFGF.

**Conclusion:**

Vitamin E supplementation for 8 weeks in the PCOS women had beneficial effects on body weight, Ang- 1, Ang-1/Ang-2 ratio, and VEGF level (Registration number: IRCT201610193140N18).

## Introduction

Polycystic ovary syndrome (PCOS) is one of the most complex endocrine disorders that causes infertility due to ovulation failure in women (1). Approximately 6-25% of women of reproductive age, are influenced by PCOS (2). The prevalence of PCOS in Iranian women was reported as 19.5% based on the Rotterdam criteria (3). The clinical symptoms of PCOS include menstrual dysfunction, hyperandrogenism, polycystic ovaries, and subfertility (2). Additionally, PCOS can cause obesity and metabolic disorders such as insulin resistance, dyslipidemia, raised levels of inflammatory factors, and endothelial dysfunction. Long-term consequences of PCOS are endometrial cancer, diabetes mellitus, hypertension, and cardiovascular disorders (4, 5). The etiology of PCOS remains largely unknown, however, there is accumulating evidence suggesting that angiogenesis dysregulation might play the main role in the pathogenesis of PCOS (4). Angiogenesis is a complex physiological process where new vessels develop from preexisting vasculature (5). Angiogenesis in the ovary is an important part of the process of the menstrual cycle (6). An essential role of the formation of the new vessels in the ovary, is the provision of nutrients and hormones for development of the corpus luteum and follicular growth (4).

Vascular endothelial growth factors (VEGFs) and angiopoietins are among the most important angiogenic markers. Other indices include basic fibroblast growth factor (bFGF) -also known as fibroblast growth factor-2 (FGF2)- and platelet-derived growth factor (PDGF). Angiopoietin1and -2 (Ang-1 and Ang-2, respectively) as well as VEGF play major roles in the regulation of angiogenesis in the ovary (4).

It was suggested that PCOS women have imbalances in angiogenic/antiangiogenic indices with partial dominance of pro-angiogenic markers. In this regard, increased ovarian expression of VEGF and bFGF has been reported in PCOS women. In addition, elevated levels of Ang-1 were shown in PCOS women compared to the healthy controls (4, 7). The abnormal alterations can cause cysts in the ovary, and disrupt and reduce ovulation rates. The recovery of proper blood vessel development in the ovaries, could improve follicular growth as well as development and ovulation among patients with PCOS (8).

Several studies have suggested that tocopherols reduce the processes of inflammation and angiogenesis (9). In addition, vitamin E levels in the blood of women with PCOS were lower than those of healthy subjects (10). Rahmani et al. (11) reported that vitamin E cosupplementation with omega-3 fatty acids, significantly regulated lipid profile and reduced oxidative stress products in PCOS women. Vitamin E and D co-supplementation was been shown to improve pregnancy outcome in PCOS women (12). In another study, vitamin E supplementation inhibited VEGF-A-mediated angiogenesis (13). In addition to the role of vitamin E in angiogenesis, some evidence indicated that vitamin E has an association with obesity (14). It seems that the mentioned effects are not due to the antioxidant mechanism of vitamin E (12).

Considering data scarcity in this subject, the present study was conducted to evaluate the effect of vitamin E on serum VEGF, bFGF, Ang-1, and Ang -2 as well as Ang-1/Ang-2 ratio in PCOS women. We hypothesized that vitamin E supplementation might have an effect on the angiogenic markers and imbalances in patients with PCOS.

## Materials and Methods

This double-blinded, placebo-controlled clinical trial was part of a lager study approved by the Ethics Committee of Tabriz University of Medical Sciences (Ethics approval No. IR.TBZMED. REC.1395.777) and registered at Iranian Registry of IRCT (IRCT201610193140N18). It was performed at the referral clinic affiliated to the Tabriz University of Medical Sciences, Tabriz, Iran from April 2017 to September 2017. The study was advertised in different clinical and therapeutic centers. For the present study, the sample size was calculated based on the results of blood VEGF concentration reported by Mondul et al. (13) by using G*Power (version 3.1.2, Germany). The number of participants was calculated as at least 16 subjects in each group. Considering dropout and to ensure a sample size sufficiently large to enable reliable estimates, we enrolled 22 and 21 subjects in vitamin E and placebo groups, respectively.

The volunteers were given more details on the study by the first author, and then, a written consent form was signed by all of the participants. The participants were able to withdraw from the study at any time.

The inclusion criteria of the study were women within the age range 20-40 years who were diagnosed with PCOS in accordance with the Rotterdam criteria (15). On the other hand, menopause, pregnancy or lactation, diabetes, having hepatic, renal, thyroid, coagulation or cardiovascular disorders, elevated levels of prolactin, smoking, alcohol consumption, fat malabsorption, receiving oral anticoagulants, ovulation induction agents or drugs affecting hormonal profile such as oral conceptive pills (OCP), or having taken antioxidant supplements or adopted a diet or a particular plan for physical activity within the last 3 months, were considered exclusion criteria (16). 

### Trial design

Trial design was parallel. Initially, forty-three PCOS women with 25 ≤ body mass index (BMI)< 35 kg/m^2^ , enrolled in to the study. The participants were randomly assigned into one of the two groups (in a 1:1 ratio), using the Random Allocation Software. The subjects in vitamin E and placebo groups received 400 IU/day vitamin E -as alpha tocopheryl acetate- (n=22), or cellulose capsules (n=21), for 8 weeks.

Vitamin E capsules were produced by Nature Made Pharmaceutical Company (USA, Batch number: 1143156) and provided by Pourateb Pharmaceutical Company (Iran). The placebo capsules were made by Barij Essence Pharmaceutical Corporation (Iran). The capsules of vitamin E and placebo were similar in size and shape. The patients and researcher were blind to allocations until the end of the study. Based on the guidelines (17), all patients received metformin at the dose of 1500 mg (500 mg 3 times daily). At the baseline of the study, the patients were asked to keep their physi cal activity and diet unchanged within the 8weeks of intervention.

### Adherence to the study

To assess the compliance, the participants were requested to bring the medication containers. All patients were monitored by a weekly phone call and encouraged to consume the supplement. Short Message Service was sent to the patients’ cell phones every day. To check the adherence to treatments, the participants were asked to bring the unused capsules. The subjects who had incomplete consumption of the drugs (less than 90% consumption) were excluded from the study.

### Evaluation of anthropometrics

Body weight (following overnight fasting) was measured by a digital scale (Seca, Hamburg, Germany) with an accuracy of ± 0.1kg. Height was measured by a non-elastic strip (Seca, Germany) with a precision of 0.1 cm. Further, BMI was calculated as weight in kilograms divided by squared height in meters. The body composition indices including body fat mass percentage (FM%), fat mass (FM), and fat free mass (FFM), were evaluated by a bioelectrical impedance analyzer (8-electrode, TanitaBC-418 MA; Tanita Co., Japan).

### Assessment of dietary intake and physical activity

Dietary intake was assessed by 24-hour recall, which was completed on three different days of the week (two weekdays and one weekend). To assess the nutrient intake of the patients, Nutritionist IV software (First Databank, CA) edited for Iranian foods, was used. To control the confounding effects of physical activity, international physical activity questionnaire-short form- (IPAQ-S) was employed for evaluation of physical activity (18). The validity and reliability of the Persian translation of IPAQ in previous studies on the Iranian populations, were tested and approved (19). We assessed physical activity and dietary intakes at the baseline and at the 8th week of intervention.

### Laboratory analysis

At the beginning and the end of the study, the patients were instructed to refer to the laboratory on days 3 to 5 of normal menstrual cycle or menstrual induced by progesterone. Fasting blood samples were obtained from the participants. To separate the serum, centrifugation at 1200 rpm for 12 minutes, was done. The samples were kept at -80°C for subsequent experiments (20). Serum levels of Ang-1 and Ang-2, VEGF, and bFGF were measured using the commercial Enzyme-Linked Immunosorbent Assays (ELISAs, Bioassay Technology Laboratory, Shanghai Korain Biotech, China) according to the manufacturer’s instructions. Coefficients of variation of the intra-assay and inter-assay assays were less than 10%.

### Statistical methods

Data analysis was performed by an intention-totreat procedure where missing values were treated based on Last-Observation-Carried-Forward method. To identify within-group differences (pre- and postintervention), we utilized paired samples t tests. To determine between group differences, independent sample t test and Mann-Whitney U test were used for comparison of normally and abnormally distributed variables, respectively. To identify the impacts of vitamin E supplementation on anthropometric and biochemical variables, analysis of covariance adjusted for age, physical activity, BMI, and baseline values, was employed. We used Statistical Package for Social Science version 25 (SPSS Inc., Chicago, Illinois, USA) for all statistical analyses, with P<0.05 considered significant.

## Results

**Fig.1:**
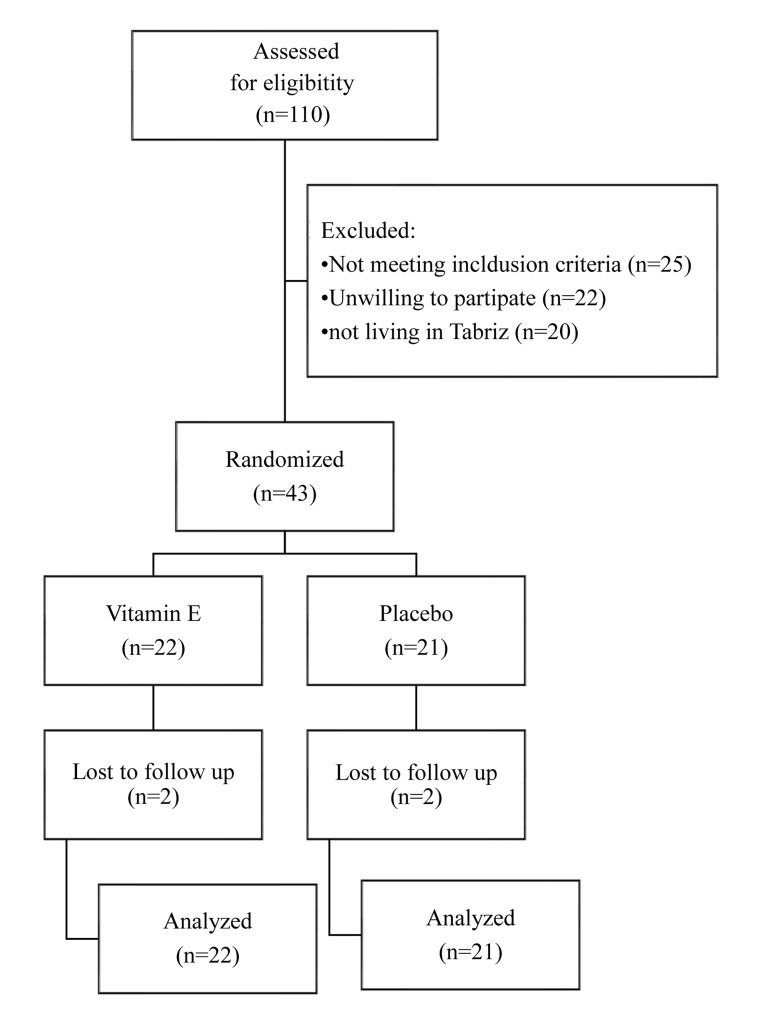
Flowchart of the study.

Four subjects dropped out the study because of pregnancy (n=1) or *in vitro* fertilization (n=3). Finally, 39 participants remained in the study. However, intention-to-treat analysis was used, so, data for 43 PCOS women were included in the final analysis (Fig .1). No major side effect was observed following taking vitamin E supplement. Hyperandrogenism (clinically) and PCOS (by ultrasound) were seen is nearly all of the subjects. Ninety percent of the participants had oligoanovulation. The compliance rate of the studied groups was more than 90%. The baseline characteristics of the participants are shown in Table 1. There were no significant differences between the two groups in terms of age, height, and physical activity. Baseline measures for follicle-stimulating hormone (FSH), and luteinizing hormone (LH) were not different between the vitamin E and placebo groups. More details on the hormonal status of the subjects are given in another published article (16).

**Table 1:** Baseline characteristics of the studied subjects in the vitamin E and placebo groups


Variables	Vitamin E n=22	Placebon=21	P value^*^

Age (Y)	27.18 ± 5.77	26.0 ± 4.53	0.68
Height (cm)	162.27 ± 6.86	159.81 ± 6.06	0.22
Physical activity (MET-minute/week)			
Low	8 (36.4)	10 (47.6)	
Moderate	11 (50)	7(52.4)	0.54^*^^*^
Vigorous	3(13.6)	4 (19)	


Data are presented as mean ± SD or n (%).*; Assessed by independent t test and **; Chisquare test, and METs; Metabolic equivalents.

### Dietary intake

Table 2 presents dietary intakes of the studied subjects. There were no significant differences in the dietary intakes of energy and nutrients between the two studied groups.

**Table 2:** Dietary intakes of the study participants throughout the study in the vitamin E and placebo groups


Variables	Vitamin E n=22	Placebo n=21	P value^*^

Energy (Kcal/day)	1698.46 ± 215.88	1745.87 ± 308.00	0.56
Carbohydrate (g/day)	214.89 ± 28.03	227.63 ± 52.57	0.32
Protein (g/day)	66.65 ± 13.66	56.9 ± 12.77	0.36
Fat (g/day)	68.05 ± 15.75	63.57 ± 18.47	0.39
SFAs (g/day)	16.09 ± 7.71	15.28 ± 7.48	0.73
PUFAs (g/day)	13.37 ± 5.89	12.69 ± 6.66	0.72
MUFAs (g/day)	17.2 ± 6.3	16.94 ± 6.59	0.89
Cholesterol (mg/day)	194.98 ± 58.55	206.21 ±65.66	0.56
Fiber (g/day)	20.73 ± 4.79	20.31 ± 4.71	0.77
Vitamin E (mg/day)	6.09 ± 3.1^*^^*^	6.85 ± 3.23	0.47
Vitamin A (RE/d)	440.12 ± 81.93	422.65 ± 132.51	0.60
Vitamin C (mg/d)	66.45 ± 12.05	73.80 ± 20.09	0.15
Selenium (µg/d)	43.39 ± 4.26	48.29 ± 20.80	0.30
Zinc (mg/day)	5.23 ± 1.34	5.21 ± 0.99	0.94


Data are presented as mean ± SD. SFA; Saturated fatty acid, PUFA; Polyunsaturated fatty acid, MUFA; Monounsaturated fatty acid, *; Assessed by independent t test, and **; Vitamin E level is estimated based only on dietary consumption, in the absence of the study supplement.

### Anthropometric measurements

No significant difference was found at the baseline of the study in the assessed anthropometric indices except for FM which was significantly higher in the vitamin E group. In within-group analysis, all assessed anthropometric indices had significant changes in the vitamin E supplemented group (P<0.01). In betweengroups comparisons, except for FFM, the assessed anthropometric indices were reduced in the vitamin E-supplemented group compared to the placebo group (Table 3).

**Table 3:** Baseline and 8 weeks after intervention values of the anthropometric indices in the vitamin E and placebo groups


Variables	Vitamin E n=22	Placebo n=21	P value

Weight (kg)			
Before	76.95 ± 10.61	73.23 ± 7.58	0.19^b^
After	75.96 ± 10.3	73.29 ± 7.3	0.01^c^
P value^a^	0.003	0.82	
BMI (kg/m^2^)			
Before	29.45 ± 5.35	28.80 ± 3.71	0.64^b^
After	29.07 ± 5.16	28.83 ± 3.70	0.01^c^
P value^a^	0.003	0.75	
FM (kg)			
Before	29.57 ± 4.41	27.08 ± 3.55	0.05^b^
After	28.25 ± 4.45	26.87 ± 3.84	0.001^c^
P value^a^	0.001	0.34	
FFM (kg)			
Before	46.86 ± 4.26	44.92 ± 2.73	0.08^b^
After	47.57 ± 4.14	44.86 ± 2.93	0.22^c^
P value^a^	0.004	0.83	
FM (%)			
Before	36.51 ± 5.54	34.24 ± 2.85	0.09^b^
After	34.85 ± 5.38	33.89 ± 2.85	0.001^c^
P value^a^	0.001	0.90	


Data are presented as mean ± SD. BMI; Body mass index, FM; Fat mass, FFM; Fat free mass, ^a^ ; P value for paired t test, ^b^; P value for Independent sample t test, and^ c^ ; P value for ANCOVA: adjusted for total calorie intake, dietary vitamin E intake, age, physical activity and baseline values.

### Angiogenic markers

The effects of vitamin E on angiogenic indices are shown in Table 4. The basal values of the angiogenic markers were not different between the two groups. In within-group analysis, VEGF, bFGF, Ang-1, and Ang-1/Ang-2 all had significant reductions in the vitamin E-supplemented group. In between-group comparisons, after adjustment for age, BMI, physical activity, total calorie intake, dietary vitamin E intake, and baseline values, supplementation with vitamin E had significant effects on VEGF, Ang-1,andAng-1/Ang-2 ratio (P=0.01, P=0.001 and P=0.03, respectively).

**Table 4:** Baseline and 8 weeks after intervention values of the serum angiogenic markers in the vitamin E and placebo groups


Variables	Vitamin E n=22	Placebo n=21	P value

VEGF (pg/mL)			
Before	733.15 (678.03, 1332.15)	423.40 (240.45, 1879.55)	0.96^b^
After	329.85 (290.00, 1381.06)	420.00 (274.15, 1628.72)	0.01^c^
P value^a^	0.005	0.48	
bFGF (pg/mL)			
Before	345.20 (305.99, 631.65)	370.10 (301.35, 590.45)	0.76^b^
After	314.18 (231.95, 318.80)	386.00 (303.7, 642.75)	0.24^c^
P value^a^	0.003	0.66	
Ang-1 (pg/mL)			
Before	1627.16 (1381.54, 2814.50)	1461.80 (1175.90, 1811.05)	0.28^b^
After	864.80 (645.90, 1627.16)	1305.01 (1305.01, 1774.45)	0.001^c^
P value^a^	0.001	0.87	
Ang-2 (pg/mL)			
Before	427.35 (247.78, 590.10)	432.49 (238.00, 493.45)	0.68^b^
After	436.65 (250.70, 554.70)	410.91 (332.75, 410.91)	0.83^c^
P value^a^	0.81	0.49	
Ang-1:Ang-2			
Before	3.44 (2.97, 5.27)	3.41 (2.72, 5.12)	0.49^b^
After	2.63 (1.46, 3.74)	3.52 (3.17, 4.78)	0.03^c^
P value^a^	0.03	0.61	


Ang-1; Angiopoietin-1, Ang-2; Angiopoietin-2, VEGF; Vascular endothelial growth factor, bFGF; Basic fibroblast growth factor, ^a^ ; P value for Wilcoxon test, ^b^; P value for Mann-Whitney U-test, and ^c^ ; P value for ANCOVA: adjusted for total calorie intake, dietary vitamin E intake, age, physical activity and baseline values. Data are shown as median (25^th^, 75^th^).

## Discussion

The present study was conducted to investigate the effect of vitamin E supplementation on the angiogenic markers in patients with PCOS. As far as we know, the present clinical trial is the first to examine the effects of vitamin E supplementation on serum angiogenic markers and anthropometric parameters in patients with PCOS. The results of this study revealed a significant reduction in weight and fat mass after eight weeks of supplementation with vitamin E among patients with PCOS. Both groups had lower energy intakes than daily estimated energy requirements (EER) for moderately active women. Low energy intake is considered a way of weight reduction, so, it is possible that the study subjects had reduced their calorie intakes for weight reduction. Only in the vitamin E group, weight reduction was significant. Few studies had assessed the effects of vitamin E supplementation on body composition components. There is some evidence about an inverse association between serum vitamin E concentration and adiposity (21). It was found that vitamin E is involved in the expression of some genes, like as leptin and peroxisome proliferator-activated receptor-γ (PPARγ), which are related to the glucose and lipid metabolism (14, 22). Leptin regulates food intake and energy balance thus plays a key role in the regulation of body fat mass (14). PPARγ is an adipogenic factor and acts as a regulator of adipogenesis (23). Increased PPARγ activity may have a positive effect on body weight gain and FM (22). Vitamin E down-regulates the expression of PPARγ (24).

Our study results indicated that vitamin E significantly lowered serum Ang-1 levels, while no change was observed in Ang-2 concentration in PCOS women. There is some evidence on angiopoietin disturbances in PCOS women. Scotti et al. (7) investigated angiopoietins of follicular fluids and reported an increase in Ang-1 but no changes in Ang-2.

In our literature review, there were no studies on vitamin E effects on the Ang-1 levels. The probable mechanism of reducing Ang-1 level by vitamin E may be linked with the reactive oxygen species (ROS). The increasing effects of ROS on the level, signaling and biological effects of Ang1 were shown. Vitamin E has antioxidative properties, so, by scavenging of ROS, it decreases ROS and therefore, Ang-1 levels (25, 26).

In our study, the Ang-1/Ang-2 ratio was decreased. Restoration of the increased level of Ang-1/Ang-2 enhances vascular progression, which in turn, promotes proper follicular evolution and increased ovulation (27). The exact mechanism(s) by which vitamin E exerts these regulatory effects are still unknown, though some possible mechanisms have been proposed. It was stated that oxidants stimulate angiogenesis while antioxidants counteract angiogenesis (28). In addition, tocopherols exert their anti-angiogenic and anti-proliferative effects through preventing signaling and activation of PI3K/PDK/ Akt signaling pathway, and inhibiting tube formation of endothelial cells (29).

Our study suggested a lowering effect for vitamin E intake on VEGF in PCOS women. There is some evidence indicating VEGF roles in the pathophysiology of PCOS (4, 30). VEGF, through neovascularization in the ovaries of PCOS patients, supports the increase in ovarian mass. Elevated levels of VEGF have been reported in women with PCOS (31). In addition, endocrine gland-VEGF, as an endothelial cell mitogen, has been shown to be over expressed in the PCOS patients’ ovaries (32). Many studies assessed the effects of vitamin E on VEGF. These studies showed different effects for tocopherol on VEGF expression and angiogenesis (33, 34). It seems that the effects are dependent on the phosphorylation status of α-tocopherol (35). In an *in vitro* study, phosphorylated α-tocopherol (αTP) stimulated VEGF generation, while non-phosphorylated (αT) form did not (36). This is the outcome of PI3K/Akt signaling pathway stimulation or inhibition. Tocopherol phosphorylation and dephosphorylation may indirectly influence proangiogenic or anti-angiogenic activities. Creation of αTP in vivo probably describes pro-angiogenic effects of vitamin E. Placenta creation, inhibition of ischemia/ reperfusion injury in the brain or cardiovascular system and promotion of wound healing are pro-angiogenic activities. Further, the pro-angiogenic ability of αTP is important in terms of expansion of solid tumors (37).

Our study did not demonstrate the effect of vitamin E on the bFGF in PCOS patients. In contrast to VEGF, little is known about agents influencing bFGF. bFGF has important functions in the ovarian angiogenesis. bFGF is a follicle-stimulating hormone, expressed in theca and granulosa cells leading to promotion of follicular growth and managing its activity (38). bFGF enhances angiogenesis by different mechanisms including stimulation of endothelial cell reproduction, chemotaxis and formation of matrix repairing enzymes such as plasminogen activator and collagenase (39). bFGF has been associated with obesity which is a common characteristic of PCOS. In addition, Artini et al. (40) reported higher levels of bFGF in serum and follicular fluids of patients with PCOS. Thus, correction of bFGF alterations in the biological fluids of PCOS women should be further examined.

Our study had some limitations. The most important limitation of the study was lack of measurement of vitamin E concentration at the baseline and at the end of study. In our study, the duration of the disease was not assessed. Another limitation was the small sample size. We could not provide a sonographic evaluation of ovarian masses at follow-up. Additionally, self-reported dietary intakes -which have the probability of under/over-reporting- and short duration of the intervention were other limitations for our study

## Conclusion

In patients with PCOS, vitamin E supplementation has useful effects on some anthropometric measurements and Ang-1, VEGF and Ang-1/Ang-2 ratio in blood. These findings suggest possible beneficial effects for vitamin E on PCOS. Concerning our study limitations, further studies are recommended to explore the potential effects of vitamin E in the management of angiopoietins disturbances among PCOS patients.
